# Immunohistochemical analysis of stem cells from human exfoliated deciduous teeth seeded in carbonate apatite scaffold for the alveolar bone defect in Wistar rats ( *Rattus novergicus*)

**DOI:** 10.12688/f1000research.25009.2

**Published:** 2020-12-02

**Authors:** Tania Saskianti, Alexander Patera Nugraha, Chiquita Prahasanti, Diah Savitri Ernawati, Ketut Suardita, Wibi Riawan

**Affiliations:** 1 Pediatric Dentistry Department, Faculty of Dental Medicine, Universitas Airlangga, Surabaya, 60132, Indonesia; 2 Orthodontics Department, Faculty of Dental Medicine, Universitas Airlangga, Surabaya, 60132, Indonesia; 3 Periodontology Department, Faculty of Dental Medicine, Universitas Airlangga, Surabaya, 60132, Indonesia; 4 Oral Medicine, Faculty of Dental Medicine, Universitas Airlangga, Surabaya, 60132, Indonesia; 5 Conservative Dentistry Department, Faculty of Dental Medicine, Universitas Airlangga, Surabaya, 60132, Indonesia; 6 Biomolecular Biochemistry, Faculty of Medicine, Brawijaya University, Malang, Indonesia

**Keywords:** Bone Defect Socket, Carbonate Apatite Scaffold, Medicine, Osteogenic Ability, Stem Cell from Human Exfoliated Deciduous Teeth

## Abstract

**Background**: Stem cells from human exfoliated deciduous teeth (SHED) seeded in carbonate apatite scaffold (CAS) may have multiple functions that could be used to regenerate the alveolar bone defects. The purpose of this study is to examine the ability of SHED and CAS in alveolar bone defects using an immunohistochemical analysis.

**Methods**: ten three-month-old healthy male Wistar rats *(R. novergicus*) that weighed between 150–250 grams (g) were used as animal models. A simple blind random sampling method was used to select the sample that was assigned to the study group for CAS and SHED seeded in CAS (n=5). The animal study model of the alveolar bone was established by extracting the anterior mandible teeth. Rodent anesthesia was applied to relieve the pain during the procedure for all test animals. Immunohistochemistry was performed after seven days to facilitate the examination of the receptor activator of NF-κβ ligand (RANKL), osteoprotegrin (OPG), transforming growth factor-β (TGF-β), vascular endothelial growth factor (VEGF), runt-related transcription factor 2 (RUNX2), alkaline phosphatase (ALP), osteocalcin, and osteopontin expression. The data was analyzed using the unpaired t-test (p<0.01) and Pearson’s correlation test (p<0.05).

**Results**: The OPG, RUNX2, TGF-β, VEGF, ALP, osteocalcin, and ostepontin expressions were higher in SHED seeded in CAS than CAS only with a significant difference between the groups (p<0.01). Furthermore, the RANKL expression was lower in SHED seeded in CAS compared to CAS only. There was a strong reverse significant correlation between OPG and RANKL expression (p<0.05).

**Conclusion**s: The number of osteogenic marker expressing cells, such as OPG, RUNX2, TGF-β, VEGF, ALP, osteocalcin, and ostepontin, increased. However, RANKL expression in the alveolar bone defects that were implanted with SHED seeded in CAS did not increase after seven days.

## Introduction

Periodontitis is the second most prevalent oral disease after dental caries ^1^. Approximately 743 million people globally suffer from periodontitis, and this figure has increased by 57.3% over the last ten years ^2, 3^. Globally, the losses that are due to reduced productivity caused by severe periodontitis are estimated to be 53.99 million United States (US) dollars annually ^3, 4^. Periodontitis is common in Indonesia ^5^. A previous study that was conducted by the Health Ministry of Republic of Indonesia in Basic Health Research ( *Riset Kesehatan Dasar* or RISKESDA) in 2018 showed that there was a 74.1% prevalence of periodontitis ^6^. The rate of periodontitis varies in each country, but together with dental caries, periodontitis is the main reason for tooth loss in adults ^1, 7^. Low socio-economic conditions in certain populations will increase the prevalence and extent of the tooth loss, which can result in an alveolar bone defect due to the limited access to dental treatment ^8^. Tooth extraction can lead to alveolar bone resorption and the destruction of the alveolar bone components ^9^. The resorption of the alveolar bone or a reduction in the jawbone dimensions might occur ^9– 11^. The presence of periodontal disease, irrational or traumatic dental extraction, periapical root fractures or alveolectomies during dental extractions are considered risk factors for or the etiology of an alveolar bone defect ^12^. An alveolar bone defect can be problematic for dental rehabilitation due to the placement of dental prosthethics ^13^. Osseointegrated of dental implants with sufficient initial stability requires adequate bone quality and quantity. Moreover, it is suggested that socket preservation is performed to enhance the success of the osseointegrated dental implants ^14^.

In the dental medicine field, the management and rehabilitation of alveolar bone defects has long been viewed as a challange ^15^. To overcome alveolar bone defects, dentists must consider bone grafting surgeries for socket preservation to obtain an adequate bone density, volume, quality, and geometry for the implant placement. This will enable osseointegration of the dental implant ^15^.

There have been many attempts to overcome alveolar bone defects, such as bone grafts, platelet rich fibrin (PRF), mesenchymal stem cells, hematopoetic stem cells, and herbal medicine ^16– 23^. Bone grafts are still not effective; therefore, alternative tissue engineering approaches are required ^24^.

 The current most promising treatment for an alveolar bone defect is through regenerative medicine, which uses tissue engineering. This tissue engineering involves three components, and is therefore referred to as triad tissue engineering: growth factors, stem cells, and a scaffold ^25, 26^. Mesenchymal stem cells (MSCs) can differentiate into various cells, such as osteogenic, adipogenic and chondrogenic differentiations ^27^. The oral cavity provides a rich source of MSCs. MSCs, such as gingiva mesenchymal stem cells (GMSCs), dental pulp stem cells (DPSCs), and stem cells from human exfoliated deciduous teeth (SHED), can be easily isolated and obtained from the oral cavity tissue using minimally invasive procedures comparted to those needed for bone marrow mesenchymal stem cells (BM-MSCs) ^28– 31^.

SHED is one of the MSCs from the oral cavity that can be used to regenerate damaged tissue, such as an alveolar bone defect ^24^. SHED is capable of differentiating and proliferating. Moreover, to optimally facilitate SHED proliferation, cell growth, and differentiation, a biocompatible cell carrier or scaffold is necessary ^32^. Carbonate apatite is a biomaterial that is commonly used as a scaffold. Carbonate apatite has been clinically proven to be a good bone scaffold for the regenerative medicine ^33^. The study about combination of SHED and CAS ameliorate alveolar bone defect post tooth extraction is still limited. The hypothesis of this study is that the number of osteogenic markers expressing cells, such as OPG, RUNX2, TGF-β, VEGF, ALP, osteocalcin, and ostepontin, would increase in the alveolar bone defects seven days after being implanted with SHED seeded in carbonate apatite scaffold (CAS), with the exception of the receptor activator of NF-κβ ligand (RANKL) expression. Osteocalcin, osteopontin, ALP, RUNX are the osteogenic differentiation markers of SHED. CAS can facilitate the osteogenic differentiation of SHED *in vitro*. Meanwhile, RANKL / OPG ratio are well-known as markers that can be used to predict the success of bone remodeling. Some growth factors are secreted by SHED, such as TGF-β and VEGF, which have an important role in supporting bone formation and controlling the inflammation process ^17– 24, 32– 34^. Wistar rats ( *Rattus novergicus*) were selected as the animal models because many studies have used this animal to study the effect of medication on the alveolar bone defects ^21– 24^. Additionally, these rats are not aggressive, and they are easy to handle and observe. This made them suitable animal models to induce the response of the human tissue system. Furthermore, the purpose of this study is to examine the ability of SHED and CAS in the alveolar bone defects using an immunohistochemical analysis.

## Methods

### Ethical clearance

 All experimental procedures involving animals were carried out in accordance with the guidelines from the National Health Institute on the care and use of laboratory animals to ameliorate any suffering for the animals.

### Study design

 This study was an experimental laboratory design. A post-test-only control group study design was conducted. The formulation that used to calculate the sample size in this study was sample size = 2SD ^2^(Z ^α/2^+ Zβ) ^2^/d ^2^ where the standard deviation (SD) = 1.1; Z ^α/2 ^= Z _0.05/2 _= Z _0.025_ =1.96 (from Z table) at type 1 error of 5%; Zβ = Z _0.20 = 0.842 _(from Z table) at 80% power; d = effect size = 1.94. The number of samples, which was five trial animals in each group. The sample in each group was randomly chosen by giving each trial animal a tag number. Following that, the researcher randomly chose the tag numbers.

### SHED Isolation, Culture, and Sub-Culture

 The SHED were obtained from deciduous teeth using the following criteria: #83, #73 deciduous tooth, free of caries, no root resorption, and a vital and intact pulp was obtained through tooth extraction from a healthy, 7–10 years-old pediatric patient who underwent orthodontics treatment. The healthy deciduous tooth was extracted from healthy pediatric patients undergoing orthodontics treatment performed at the Dental Hospital, Universitas Airlangga, Surabaya, Indonesia. Patient anonymity was maintained and written informed consent was obtained from the patient’s parents. Ethical approval was obtained from the Universitas Airlangga, Faculty of Dental Medicine ethics committee (171/HRECC.FODM/VIII/2017) that covered for both human sampling and the animal procedures.

 The dental pulp cavity was opened using drills under aseptic condition. The dental pulp was isolated with a broach then washed three times with phosphate-buffered saline (PBS). Dental pulp tissue was minced into small pieces (≤0.5 mm) in 10-cm culture dishes digested in a solution of 3 mg/mL collagenase type I (no cat. CLS-01, Worthington Biochem, Freehold, NJ) and 4 mg/mL Dispase® II (cat no. 42613-33-2, Sigma Aldrich, USA) for 1 h at 37°C. Dulbecco′s Modified Eagle′s Medium (cat no. D5030, Merck, US), was utilized to culture the dental pulp from the deciduous tooth. Fetal bovine serum (FBS, catcat no. F2442, Merck, US) with 20% concentration, five milimeter L-glutamine (cat no. 5030081, Gibco Invitrogen®, 25, USA), 100 U/ ml penicillin-G, 100 ug/ml streptomycin, and 100 ug/ml kanamycin (cat no. 15140163, Gibco Invitrogen®, 25, USA) was added ^34^.

 Every four days, the medium was changed to eliminate the unattached cell on the culture plate and the cells were maintained up to four passages. Phosphate Buffer Saline was used to wash the cells to eliminate debris. Trypsin-EDTA 0.05% was applied to detach the cells and transfer them onto a bigger culture plate. After the cells reached 70–80% confluence was obtained, the SHED cells in the 4 passaged were then prepared for the next step of the study ^24, 32, 34^.

### The alveolar bone defect in animal models

Ten three-month-old healthy male Wistar rats *(R. novergicus*) that weighed between 150–250 grams (g) were used as animal models and were obtained from the Research Center of Faculty of Dental Medicine, Universitas Airlangga (UNAIR) Surabaya, Indonesia. Ten wistar rats were assigned into two groups respectively; CAS group and CAS+SHED group.

 All experimental procedures involving animals were carried out in keeping with guidelines from the National Institutes of Health Guide for the Care and Use of Laboratory Animals to ameliorate any suffering of animals ^35^. The animal models were acclimatized for a week at a temperature of 21–23 °C with controlled humidity (50 ± 5%) in a 12-hour artificial light cycle (8 am to 8 pm) to help them to adapt to the same conditions, as they had various origins. All the rats were located individually in polycarbonate cages (0.90 × 0.60 × 0.60 m). Furthermore, every animal model was fed with standard pellet, and water was provided *ad libitum* with the husk replaced every three days. All animal models were routinely inspected and observed regarding their food consumption and fecal characteristics ^20^.

 Rodent anesthesia of 0.1 mL/10 grams body weight (BW) (160095, Kepro™, Netherlands), and xylazine (160096, Xyla™, Netherlands) (ketamine dose 35 mg/kg body weight and xylazine five mg/kg body weight) was administered intramuscularly on the gluteus muscle to ameliorate the pain during the procedure of inducing the alveolar bone defects on the animal models. Sterile needle holder clamps were used to extract the anterior teeth of the mandibular to induce the alveolar bone defects in the animal models ^36^.

### The Transplantation of Stem Cells from Human Exfoliated Deciduous Teeth Seeded in Carbonate Apatite Scaffold

 After the alveolar bone defect was induced, the transplantation of the SHED seeded in CAS or CAS only was performed in the afflicted area. Before being placed in a 24-well tissue culture plate and prepared for the experimental group, a 20 ml suspension of SHED at passage four to five with a density of 10 ^6^ cells was seeded into CAS (no cat AKD 20602410125, GAMACHA, Swayasa Prakarsa Company, Indonesia). The dose was determined based on the evidence from a previous *in vivo* study, which was 10 ^6^ cells per sample ^34^. To perform the interrupted suture to fix the wound after transplantation, a 5.0 suture monofilament was used ^24, 32, 34^.

Seven days post transplantation, all the animal models were terminated using an overdosed rodent anesthesia with an intravenous injection of 100 mg/kg BW (Pentobarbital, 1507002, Pubchem, USA). We used this euthanasia method to ameliorate animal suffering that arises from the termination process. After the termination of animal study, we collected the afflicted alveolar bone samples for further histological analysis. The animal model’s head was cut from the back by sterile sharp surgical scissors (metzenbaum scissors fine tips, no cat. 3565, Medesy, Maniago, Italy) and tweezer (Tweezer de bakey mini, no cat. 1007/10-TO, Medesy, Maniago, Italy), exposing the anterior of the mandible allowing the afflicted alveolar bone sample to be obtained. Before sample collection, all the animals were observed for any general toxicity probability, including edema or death, and measured had their body weight (using a digital scale, ZB22-P, Zieis®, USA). All these measurements were done by a single blinded observer. The afflicted tissue was then extracted and immersed in 10% neutral buffer formalin for fixation.

### Tissue Processing, Embedding and Sectioning

The sample was decalcified and immersed in 10% EDTA (cat no. 17892, Ajax Finechem, Thermo Fisher Scientific; Taren Point, Australia). Following that, the samples were underwent tissue processing overnight (Leica TP1020, USA), prior to embedding in molten paraffin wax (HistoCore Arcadia H - Heated Paraffin Embedding Station, Leica, USA). Sections were cut at 5 µm rotary microtome (RM2235, Leica, USA). Paraffin ribbons were flattened in a water bath at 40°C and collected onto polysine microscope slides (Thermo Scientific) prior to drying at 60°C for 16 hr (Sakura Heater, Tokyo, Japan) ^37^.

### Immunohistochemistry staining

 Immunohistochemistry staining was conducted using a 3.3'-diaminobenzidine stain kit (DAB) (cat noD7304-1SET, Sigma Aldrich, US). Antibody monoclonal (AbMo) of RANKL 1:500 dilution (cat. no sc-377079), osteoprotegrin (OPG) 1:500 dilution (cat. no sc-390518), runt-related transcription factor 2 (RUNX2) 1:500 dilution (cat. no sc-390351), transforming growth factor-β (TGF-β) 1:500 dilution (cat. no sc-130348), vascular endothelial growth factor (VEGF) 1:500 dilution (cat. no sc-7269), alkaline phosphatase (ALP) 1:500 dilution (cat. no sc-271431), osteocalcin (cat. no sc-365797)) 1:500 dilution, and ostepontin (cat. no sc-21742) 1:500 dilution were used in this study (Santa Cruz Biotechnology™, US). The observation and examination of the number of the RANKL, OPG, RUNX2, TGF-β, VEGF, ALP, osteocalcin, and ostepontin expressions in the periodontal tissue were performed manually by two observers in five perspective fields of view by utilizing Nikon H600L light microscope (Japan) at 400x magnification. We also provide 200x and 1000x magnification of each marker for context (Nikon, Japan) ^37^.

### Statistical analysis

The Statistical Package for Social Science (SPSS) 20.0 version (IBM corporation, Illinois, Chicago, United State) software was used in this study to analyze the data. To compare the significant differences between the groups in the RANKL, OPG, RUNX2, TGF-β, VEGF, ALP, osteocalcin, and ostepontin expressions, a t-test was employed (p<0.01). The OPG and RANKL expressions’ association was examined using Pearson’s correlation test (p<0.05).

## Results

**Figure 1.**
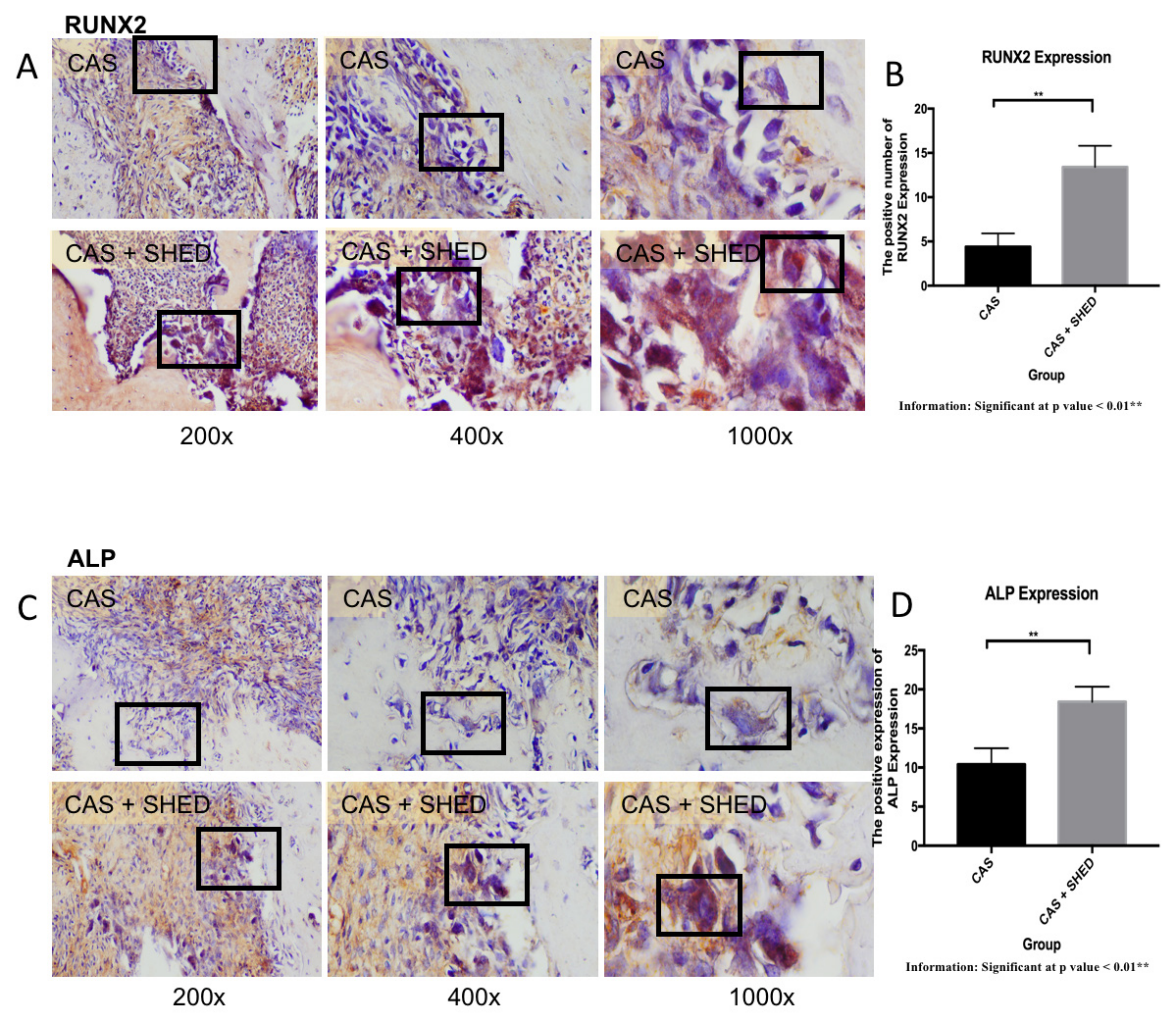
Histological sections of the Wistar rats’ ( *R. Novergicus*) afflicted periodontal tissues. Immunohistochemistry with antibody monoclonal (AbMo) and DAB were performed to examine the ( **A**) RANKL and ( **B**) OPG expressions. The positive cells were stained brown (black box) with a 200x, 400x, and 1000x magnification using a light microscope. The number of osteoblasts expressing ( **C**) RANKL and ( **D**) OPG in the alveolar bone of the rats.

The transplantation of SHED seeded in CAS or CAS only at selected doses did not lead to any general toxicity, edema, death or changes in body weight of the rats (see underlying data ^38^). The expressions of OPG, RUNX2, TGF- β, VEGF, ALP, osteocalcin, and osteopontin in SHED seeded in CAS were greater than in the CAS only group. In comparison, the RANKL expression was lower in SHED seeded in CAS compared to CAS only (see Figure 1– Figure 4 ^39– 46^). There was a significant increase in OPG, RUNX2, TGF-β VEGF, ALP, osteocalcin, and osteopontin expressions and decreased RANKL expression in SHED seeded in CAS compared to CAS only (p<0.01). There was a significant strong reverse correlation between the OPG and RANKL expressions (p<0.01) ( Table 1).

**Figure 2.**
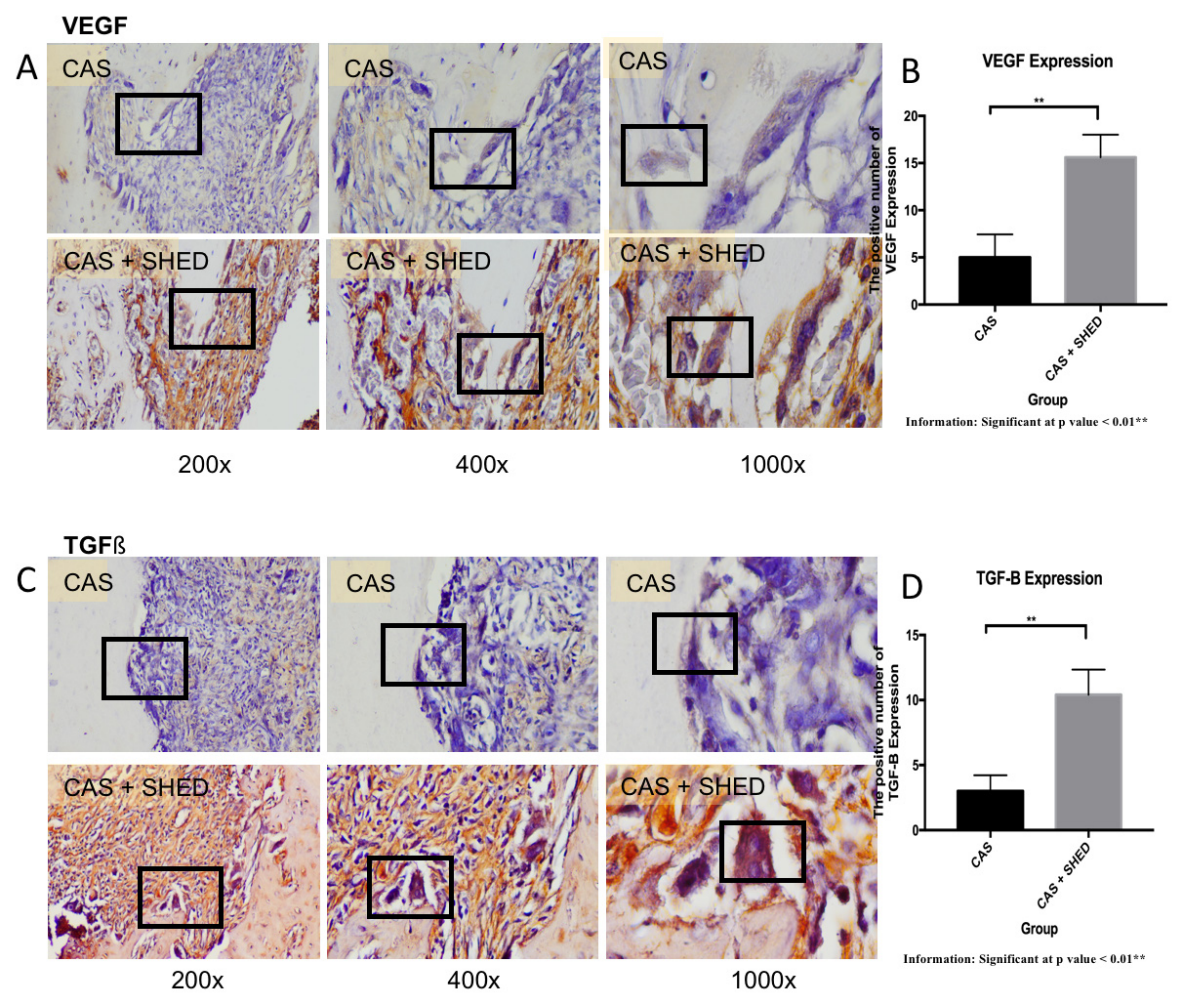
Histological sections of the Wistar rats’ ( *R. Novergicus*) afflicted periodontal tissues. Immunohistochemistry with antibody monoclonal (AbMo) and DAB were performed to examine the ( **A**) VEGF and ( **B**) TGF-β expressions. The positive cells were stained brown (black box) with 200x, 400x, and 1000x magnification using the light microscope. The number of osteoblasts expressing ( **C**) VEGF and ( **D**) TGF- β in the alveolar bone of the rats.

**Figure 3.**
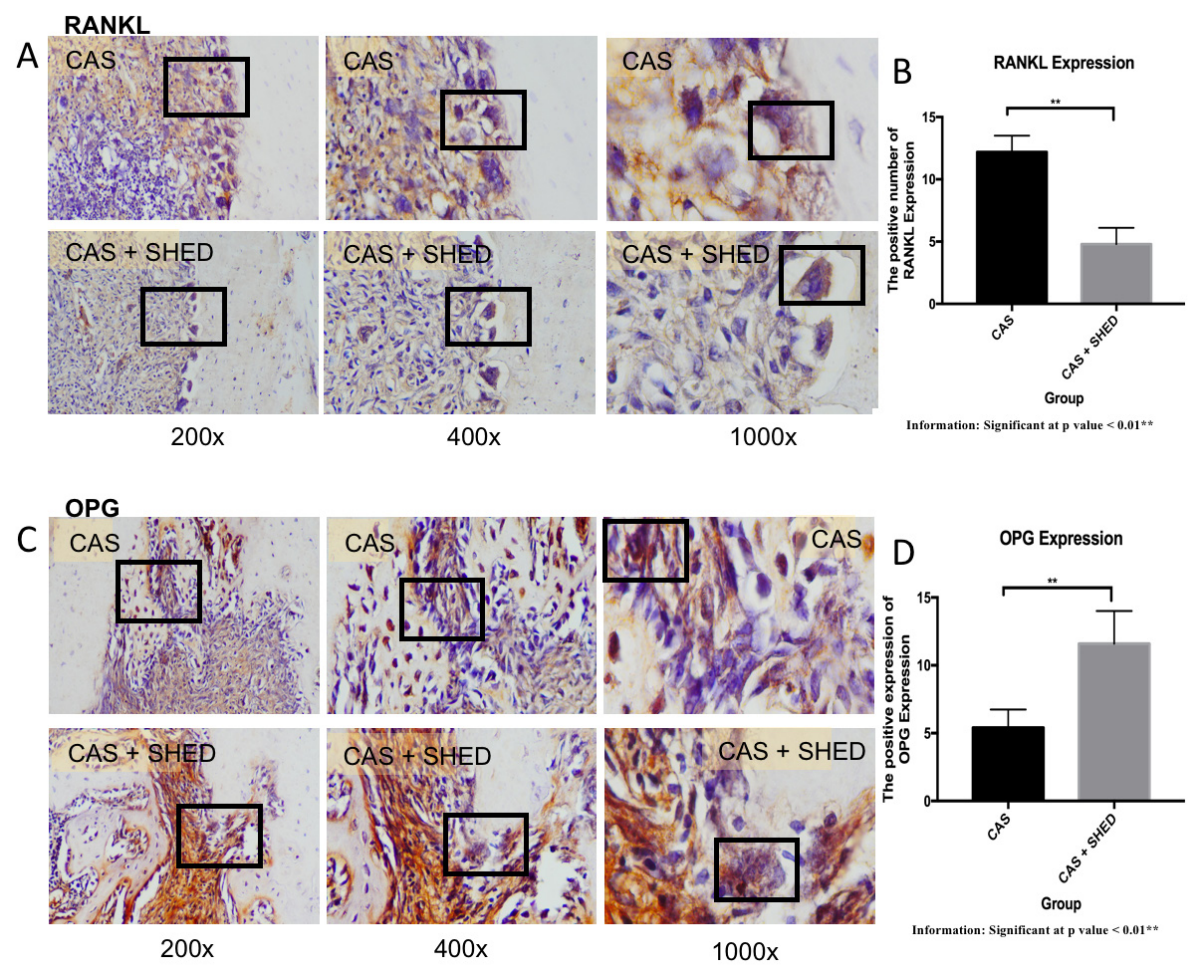
Histological sections of the Wistar rat’s ( *R. Novergicus*) afflicted periodontal tissues. Immunohistochemistry with antibody monoclonal (AbMo) and DAB were performed to examine the ( **A**) RUNX2 and ( **B**) ALP expressions. The positive cells were stained brown (black box) with 200x, 400x, and 1000x magnification using the light microscope. The number of osteoblasts expressing ( **C**) RUNX2 and ( **D**) ALP in the alveolar bone of the rats.

**Figure 4.**
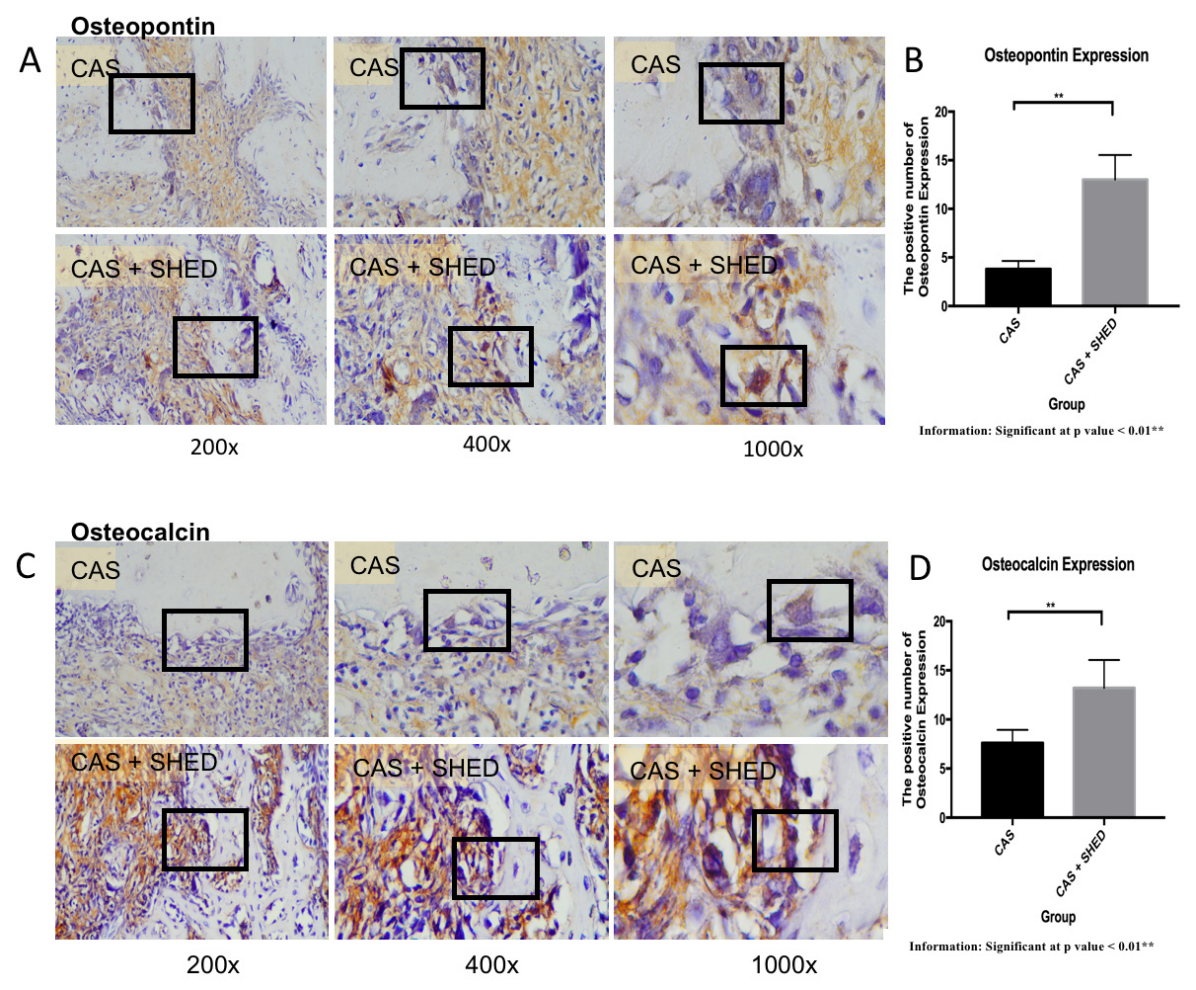
Histological sections of the Wistar rat’s ( *R. Novergicus*) afflicted periodontal tissues. Immunohistochemistry with antibody monoclonal (AbMo) and DAB were performed to examine the ( **A**) osteocalcin and ( **B**) osteopontin expressions. Positive cells were stained brown (black box) in 200x, 400x, 1000x magnification by using the light microscope. The number of osteoblasts expressing ( **C**) osteocalcin and ( **D**) osteopontin in the alveolar bone of the rats.

**Table 1.** The mean ± standard deviation, the result of the normality test and the t-test of each marker between the groups (n=5).

Group	Molecular Marker							
	Mean ± Standard Deviation							
	OPG	RANKL	TGF- β	VEGF	RUNX2	ALP	OSC	OSP
CHA	5.4 ± 0.6	4.8 ± 0.5831	3 ± 0.5477	5 ± 1.095	4.4 ± 0.6782	10.4 ± 2.073	7.64 ± 0.6	3.8 ± 0.3742
*Normality	0.21	0.421	0.146	0.146	0.86	0.23	0.201	0.314
CHA+SHED	11.6 ± 1.077	12.2 ± 0.5831	10.4 ± 0.8718	15.6 ± 1.077	13.4 ± 1.077	18.4 ± 1.949	13.2 ± 1.281	13 ± 1.14
*Normality	0.787	0.21	0.758	0.787	0.787	0.758	0.823	0.207
**Sig	0.001	0.0001	0.0001	0.0001	0.0001	0.0002	0.0042	0.0001
Pearson Correlation	-0.0801							
**Sig. Correlation	0.005							

Information: *significant at p value > 0.05; **significant at p value < 0.01. RANKL - receptor activator of NF-κβ ligand, OPG - osteoprotegrin, TGF- β - transforming growth factor-β, VEGF - vascular endothelial growth factor, RUNX2 - runt-related, transcription factor 2, ALP - alkaline phosphatase, OSC - osteocalcin, OSP osteopontin, SHED- stem cells from human exfoliated deciduous teeth, CHA - carbonate hydroxyapatite

## Discussion

Severe alveolar defect has become a problem for both the patients and clinicians, especially regarding dental implant placement and ossteointegration ^15^.

This experimental study confirms the hypothesis that the transplantation of SHED seeded in CAS could increase the number of osteogenic markers expressing cells, such as OPG, RUNX2, TGF-β, VEGF, ALP, osteocalcin, and ostepontin, but not the RANKL expression in the bone defects after seven days in comparison to the CAS group ^17– 20, 22, 32^. 

This result supports the theory that SHED possess functions that can enhance OPG to bind to RANKL, which results in the inhibited osteoclastogenesis ^34^. There is a strong reverse significant correlation between OPG and RANKL expressions in this study. The SHED with the scaffold increases the OPG expression meanwhile, decreases the RANKL expression, which is supported by the previous study by Prahasanti *et al.* ^34^

CAS plays an important role in supporting SHED proliferation and differentiation ^24, 32^. RUNX2, ALP, osteocalcin, and osteopontin are osteogenic differentiation markers of MSCs. These markers are essential and important for the analysis of osteoblastogenesis and bone regeneration ^17, 18, 20^. ALP expression increases due to the signaling bone morphogenic protein (BMP), RUNX2, osterix system, and Wnt cascade interacting with each other. The increased expression of RUNX can enhance ALP expression ^17, 18^. Several growth factors also stimulate the activation of the MSCs’ osteogenic differentiation, such as VEGF and TGF- β. TGF-β significantly increases the expression of the early-phase osteogenic differentiation marker genes ^47^. VEGF is associated with all the bone formation steps, including mesenchymal condensation ^48^. VEGF has a direct influence on the MSC osteogenic differentiation through the regulation of osteoprogenitors using the angiocrine function. VEGF recruits immune cells to the osteogenic niche ^49^.

The osteogenic microenvironment in defective alveolar bone can induce SHED to differentiate into bone cells, especially osteoblast ^24, 32, 34^. The activation of the osteorix and RUNX2 systems can stimulate the expression of osteocalcin and osteopontin ^17^. OSC is a secreted protein that is dependent on Vitamin K, a macromolecule with a role in bone mineralization ^18^. Osteopontin plays a pivotal role in bone remodeling, regulating osteoclastogenesis, osteoclast activity, and differentiation. Osteopontin maintains the bone mineral matrix inorganic components of bone, such as hydroxyapatite, Ca(PO4)(OH)2. Osteopontin, which is expressed in osteoblasts, is responsible for bone remodeling in bone homeostasis ^20^. Both osteocalcin and osteopontin are important for bone maturation because they are major non-collagenous proteins that are involved in bone matrix organization and deposition.

 Osteocalcin and osteopontin are produced during bone formation ^49^. Both of them control––either directly and/or indirectly–the mass, mineral size, and orientation ^50– 52^. Both proteins also play structural roles in the bone and determine the bone’s propensity to fracture ^53^. This is in accordance with our findings, as it states that there is a significant enhancement of the OPG, RUNX2, TGF- β, VEGF, ALP, osteocalcin, and osteopontin expressions, and the decreased RANKL expression is more significant in Group II than Group I. Bone regeneration is a complex process that requires highly orchestrated interactions between different cells and signals to form the new mineralized tissue ^54^. MSCs have the ability to differentiate into osteoprogenitors and osteoblasts, as well as to form the calcified bone matrix ^55^.

SHED have the potential to play a significant role in tissue engineering and regenerative medicine. A previous study by Nakajima *et al.* declared that SHED, in comparison to the hDPSCs or hBMSCs group, produce the largest osteoid and collagen fibers. Furthermore, SHED transplantation possess a potential and sufficient ability for bone regeneration to repair the bone defect ^56, 57^.

The limitations of this study were that the observation and evaluation were performed seven days post transplantation of SHED seeded in CAS on the animal model, and only an immunohistochemical examination was performed. Further studies will be necessary to evaluate the changes in the alveolar bone and periodontal tissue post transplantation of SHED seeded in CAS in the alveolar bone defect animal models. With a longer observation time, further studies using methods, such as qRT-PCR and/or the western blot analysis, could be conducted to estimate the expression of bone molecular markers. Future studies are also required to confirm the effective dose of the used biomaterials when it is ready to be applied in the clinical study of humans.

## Conclusion

In conclusion, the expression of OPG, RUNX2, TGF-β, VEGF, ALP, osteocalcin, and ostepontin increases significantly with treatment with SHED seeded in CAS. Moreover, the RANKL expression in the alveolar bone defect did not increase in SHED seeded in CAS as documented immunohistochemically.

## Data availability

### Underlying data

Figshare: RANKL. https://doi.org/10.6084/m9.figshare.12609986.v1 ^39^

This project contains the following underlying data:

= CAS RANKL 200x.jpg (Expression of RANKL at 200x magnification in the CAS group)
= CAS RANKL 400x.jpg (Expression of RANKL at 400x magnification in the CAS group)
= CAS RANKL 1000x.jpg (Expression of RANKL at 1000x magnification in the CAS group)
= CAS SHED RANKL 200x.jpg (Expression of RANKL at 200x magnification in the CAS SHED group)
= CAS SHED RANKL 400x.jpg (Expression of RANKL at 400x magnification in the CAS SHED group)
= CAS SHED RANKL 1000x.jpg (Expression of RANKL at 1000x magnification in the CAS SHED group)

Figshare: OPG. https://doi.org/10.6084/m9.figshare.12609983.v1 ^40^

This project contains the following underlying data:

= CAS OPG 200x.jpg (Expression of OPG at 200x magnification in the CAS group)
= CAS OPG 400x.jpg (Expression of OPG at 400x magnification in the CAS group)
= CAS OPG 1000x.jpg (Expression of OPG at 1000x magnification in the CAS group)
= CAS SHED OPG 200x.jpg (Expression of OPG at 200x magnification in the CAS SHED group)
= CAS SHED OPG 400x.jpg (Expression of OPG at 400x magnification in the CAS SHED group)
= CAS SHED OPG 1000x.jpg (Expression of OPG at 1000x magnification in the CAS SHED group)

Figshare: RUNX2. https://doi.org/10.6084/m9.figshare.12610478.v1 ^41^

This project contains the following underlying data:

= CAS RUNX2 200x.jpg (Expression of RUNX2 at 200x magnification in the CAS group)
= CAS RUNX2 400x.jpg (Expression of RUNX2 at 400x magnification in the CAS group)
= CAS RUNX2 1000x.jpg (Expression of RUNX2 at 1000x magnification in the CAS group)
= CAS SHED RUNX2 200x.jpg (Expression of RUNX2 at 200x magnification in the CAS SHED group)
= CAS SHED RUNX2 400x.jpg (Expression of RUNX2 at 400x magnification in the CAS SHED group)
= CAS SHED RUNX2 1000x.jpg (Expression of RUNX2 at 1000x magnification in the CAS SHED group)

Figshare: TGF-Beta. https://doi.org/10.6084/m9.figshare.12610487.v1 ^42^

This project contains the following underlying data:

= CAS TGF-β 200x.jpg (Expression of TGF-β at 200x magnification in the CAS group)
= CAS TGF-β 400x.jpg (Expression of TGF-β at 400x magnification in the CAS group)
= CAS TGF-β 1000x.jpg (Expression of TGF-β at 1000x magnification in the CAS group)
= CAS SHED TGF-β 200x.jpg (Expression of TGF-β at 200x magnification in the CAS SHED group)
= CAS SHED TGF-β 400x.jpg (Expression of TGF-β at 400x magnification in the CAS SHED group)
= CAS SHED TGF-β 1000x.jpg (Expression of TGF-β at 1000x magnification in the CAS SHED group)

Figshare: VEGF. https://doi.org/10.6084/m9.figshare.12610484.v1 ^43^

This project contains the following underlying data:

= CAS VEGF 200x.jpg (Expression of VEGF at 200x magnification in the CAS group)
= CAS VEGF 400x.jpg (Expression of VEGF at 400x magnification in the CAS group)
= CAS VEGF 1000x.jpg (Expression of VEGF at 1000x magnification in the CAS group)
= CAS SHED VEGF 200x.jpg (Expression of VEGF at 200x magnification in the CAS SHED group)
= CAS SHED VEGF 400x.jpg (Expression of VEGF at 400x magnification in the CAS SHED group)
= CAS SHED VEGF 1000x.jpg (Expression of VEGF at 1000x magnification in the CAS SHED group)

Figshare: ALP. https://doi.org/10.6084/m9.figshare.12610493.v1 ^44^

This project contains the following underlying data:

= CAS ALP 200x.jpg (Expression of ALP at 200x magnification in the CAS group)
= CAS ALP 400x.jpg (Expression of ALP at 400x magnification in the CAS group)
= CAS ALP 1000x.jpg (Expression of ALP at 1000x magnification in the CAS group)
= CAS SHED ALP 200x.jpg (Expression of ALP at 200x magnification in the CAS SHED group)
= CAS SHED ALP 400x.jpg (Expression of ALP at 400x magnification in the CAS SHED group)
= CAS SHED ALP1000x.jpg (Expression of ALP at 1000x magnification in the CAS SHED group)

Figshare: Osteocalcin. https://doi.org/10.6084/m9.figshare.12610481.v1 ^45^

This project contains the following underlying data:

= CAS osteocalcin 200x.jpg (Expression of osteocalcin at 200x magnification in the CAS group)
= CAS osteocalcin 400x.jpg (Expression of osteocalcin at 400x magnification in the CAS group)
= CAS osteocalcin 1000x.jpg (Expression of osteocalcin at 1000x magnification in the CAS group)
= CAS SHED osteocalcin 200x.jpg (Expression of osteocalcin at 200x magnification in the CAS SHED group)
= CAS SHED osteocalcin 400x.jpg (Expression of osteocalcin at 400x magnification in the CAS SHED group)
= CAS SHED osteocalcin 1000x.jpg (Expression of osteocalcin at 1000x magnification in the CAS SHED group)

Figshare: Ostepontin. https://doi.org/10.6084/m9.figshare.12610490.v1 ^46^

This project contains the following underlying data:

= CAS osteopontin 200x.jpg (Expression of osteopontin at 200x magnification in the CAS group)
= CAS osteopontin 400x.jpg (Expression of osteopontin at 400x magnification in the CAS group)
= CAS osteopontin 1000x.jpg (Expression of osteopontin at 1000x magnification in the CAS group)
= CAS SHED osteopontin 200x.jpg (Expression of osteopontin at 200x magnification in the CAS SHED group)
= CAS SHED osteopontin 400x.jpg (Expression of osteopontin at 400x magnification in the CAS SHED group)
= CAS SHED osteopontin 1000x.jpg (Expression of osteopontin at 1000x magnification in the CAS SHED group)

Figshare: Raw Data Bone Molecular Markers. https://doi.org/10.6084/m9.figshare.12610499.v1 ^58^

This project contains the following underlying data:

= Raw Data Molecular Marker.xlsx (The raw data of molecular markers examined by means of IHC analysis)

Figshare: Animal Body Weight. https://doi.org/10.6084/m9.figshare.12610502.v1 ^38^

This project contains the following underlying data:

= Animal Body Weight.xlsx (Animal Body Weight, pre and post test)

Data are available under the terms of the Creative Commons Attribution 4.0 International license (CC-BY 4.0).
