# Supplementary material for: Immunohistochemical analysis of stem cells from human exfoliated deciduous teeth seeded in carbonate apatite scaffold for the alveolar bone defect in Wistar rats ( Rattus novergicus)
Source: F1000Res. 2020 Dec 2;9:1164. Originally published 2020 Sep 22. [Version 2] doi: 10.12688/f1000research.25009.2 (PMC7721066; doi:10.12688/f1000research.25009.2)
Supplement: Peer review history

# Peer review: Reviewer response for version 2

**DOI:** 10.5256/f1000research.31029.r75620

**Published:** 2020-12-03

**Authors:** Niswati Fatmah Rosyida

**Affiliations:** 1 Department of Orthodontics, Faculty of Dentistry, Universitas Gadjah Mada, Yogyakarta, Indonesia

The authors have revised the article based on the my comments before. Therefore the article can be approved.

Is the work clearly and accurately presented and does it cite the current literature?

Yes

If applicable, is the statistical analysis and its interpretation appropriate?

Yes

Are all the source data underlying the results available to ensure full reproducibility?

Yes

Is the study design appropriate and is the work technically sound?

Yes

Are the conclusions drawn adequately supported by the results?

Yes

Are sufficient details of methods and analysis provided to allow replication by others?

Yes

Reviewer Expertise:

Biomaterial in Orthodontic, Biological Tooth Movement

I confirm that I have read this submission and believe that I have an appropriate level of expertise to confirm that it is of an acceptable scientific standard.

# Peer review: Reviewer response for version 1

**DOI:** 10.5256/f1000research.27586.r72008

**Published:** 2020-11-03

**Authors:** Lidia Audrey Rocha Valadas

**Affiliations:** 1 Postgraduate Program in Development and Technological Innovation in Drugs, School of Pharmacy, Federal University of Ceará, Fortaleza, Brazil

In general the main topic of the manuscript is ambitious and interesting. The paper is well designed and supported with updated references. The introduction is written in clear language, covering a large number of relevant issues. Methods show all details of the methodology used to perform the research but I suggest to provide more details about the sample. Please provide the number and place of approval of your project's ethical opinion. Results are precise and Discussion supports them.

Is the work clearly and accurately presented and does it cite the current literature?

Yes

If applicable, is the statistical analysis and its interpretation appropriate?

Yes

Are all the source data underlying the results available to ensure full reproducibility?

Yes

Is the study design appropriate and is the work technically sound?

Yes

Are the conclusions drawn adequately supported by the results?

Yes

Are sufficient details of methods and analysis provided to allow replication by others?

Yes

Reviewer Expertise:

NA

I confirm that I have read this submission and believe that I have an appropriate level of expertise to confirm that it is of an acceptable scientific standard.

# Peer review: Reviewer response for version 1

**DOI:** 10.5256/f1000research.27586.r71798

**Published:** 2020-10-08

**Authors:** Niswati Fatmah Rosyida

**Affiliations:** 1 Department of Orthodontics, Faculty of Dentistry, Universitas Gadjah Mada, Yogyakarta, Indonesia

The authors of this study have investigated the ability of SHED and CAS in alveolar bone using immunohistochemical analysis.

 Based on the study results, the authors have concluded that all the variables increased on Day 7, while RANK expression decreased. The results are certainly interesting.

 The paper is very well organized, easy to follow, having a novelty and impact.

 Minor concerns about the study are listed as follows:

**Introduction**

1. Please reduce the explanation about periodontitis as a causative factor on the bone defect, moreover this study does not use the periodontitis model.
2. Please add the information about the problem or the success of the SHED and CAS combination from current or past research.

**Method**

1. There is no information regarding the experimental animal grouping. The authors should mention it.
2. Please explain why there is no control group without treatment SHED in CAS, or SHED group only.

**Discussion**

1. There was no discussion why the histological analysis only was done only on 7 day ater treatment, what the reason?
2. CAS have proved induce the healing from the previous study. The success of this formulation is caused by the combination of SHED and CAS, but in the discussion, there is lack of CAS role. Please add the explanation.

Is the work clearly and accurately presented and does it cite the current literature?

Yes

If applicable, is the statistical analysis and its interpretation appropriate?

Yes

Are all the source data underlying the results available to ensure full reproducibility?

Yes

Is the study design appropriate and is the work technically sound?

Yes

Are the conclusions drawn adequately supported by the results?

Yes

Are sufficient details of methods and analysis provided to allow replication by others?

Yes

Reviewer Expertise:

Biomaterial in Orthodontic, Biological Tooth Movement

I confirm that I have read this submission and believe that I have an appropriate level of expertise to confirm that it is of an acceptable scientific standard, however I have significant reservations, as outlined above.

# Response

**Published:** 2020-11-25

**Authors:** Alexander Patera Nugraha

**Affiliations:** Faculty of Dental Medicine, Universitas Airlangga, Indonesia

The authors thank the Honorable Reviewer 1 for her excellent review of the paper and in-depth suggestions made to improve the quality of the paper. Followings are the responses to the suggestions of the Honorable Reviewer. The valuable suggestions and corrections are incorporated cautiously, and the paper is made clearer while revising the manuscript.

**Introduction**

 Please reduce the explanation about periodontitis as a causative factor on the bone defect, moreover this study does not use the periodontitis model. * Please kindly check the new version of our paper. We have been reduced the explanation about periodontitis as a causative factor on the bone defect due to we used the tooth extraction induce alveolar bone defect model in rats. Please add the information about the problem or the success of the SHED and CAS combination from current or past research.

 *This study novely was the transplantation of SHED seeded in CAS could increase the number of osteogenic markers expressing cells, such as OPG, RUNX2, TGF-β, VEGF, ALP, osteocalcin, and ostepontin, but not the RANKL expression in the bone defects after seven days in comparison to the CAS group. The previous researchs about combination of SHED and CAS ameliorate alveolar bone defect post tooth extraction is still limited. 

**Method**

 There is no information regarding the experimental animal grouping. The authors should mention it.

 *Ten wistar rats were assigned into two groups respectively; CAS group and CAS+SHED group. 

 Please explain why there is no control group without treatment SHED in CAS, or SHED group only.

 *The limitations of this study were that the observation and evaluation were performed on transplantation of SHED seeded in CAS and CAS only on the animal model. **Discussion** There was no discussion why the histological analysis only was done only on 7 day after treatment, what the reason?

 *The limitations of this study were that the observation and evaluation were performed seven days post transplantation of SHED seeded in CAS on the animal model, and only an immunohistochemical examination was performed. Further studies will be necessary to evaluate the changes in the alveolar bone and periodontal tissue post transplantation of SHED seeded in CAS in the alveolar bone defect animal models. With a longer observation time, further studies using methods, such as qRT-PCR and/or the western blot analysis, could be conducted to estimate the expression of bone molecular markers. 

 CAS have proved induce the healing from the previous study. The success of this formulation is caused by the combination of SHED and CAS, but in the discussion, there is lack of CAS role. Please add the explanation.

 CAS plays an important role in supporting SHED proliferation and differentiation. The osteogenic microenvironment in defective alveolar bone may induce SHED seeded in CAS to differentiate into bone cells, especially osteoblast.
